# Crystal structure of 1-(5-bromo-1-benzo­furan-2-yl)ethanone oxime

**DOI:** 10.1107/S205698901501751X

**Published:** 2015-09-26

**Authors:** G. Krishnaswamy, P. Krishna Murthy, R. Nivedita Desai, P. A. Suchetan, D. B. Aruna Kumar

**Affiliations:** a Dept. of Studies and Research in Chemistry, University College of Science, Tumkur University, Tumkur 572103, India

**Keywords:** crystal structure, 1-(5-bromo­benzo­furan-2-yl) ethanone oxime, hydrogen bonding, π–π stacking inter­actions

## Abstract

The title compound, C_10_H_8_BrNO_2_, is almost planar (r.m.s. deviation for the non-H atoms = 0.031 Å) and the conformation across the C=N bond is *trans*. Further, the O atom of the benzo­furan ring is *syn* to the N atom of the oxime group. In the crystal, inversion dimers linked by pairs of O—H⋯N hydrogen bonds generate *R*_2_^2^(6) loops. Very weak aromatic π–π stacking inter­actions [centroid–centroid separations = 3.9100 (12) and 3.9447 (12) Å] are also observed.

## Related literature   

For the various biological activities of the benzo­furan moiety, see: Rida *et al.* (2006 ▸); Manna *et al.* (2010 ▸); Patil *et al.* (2010 ▸); Patel *et al.* (2006 ▸). For the anti­fungal activity of (benzo­furan-2-yl) keoximes, see: Demirayak *et al.* (2002 ▸). For related structures, see: Aruna Kumar *et al.* (2014 ▸).
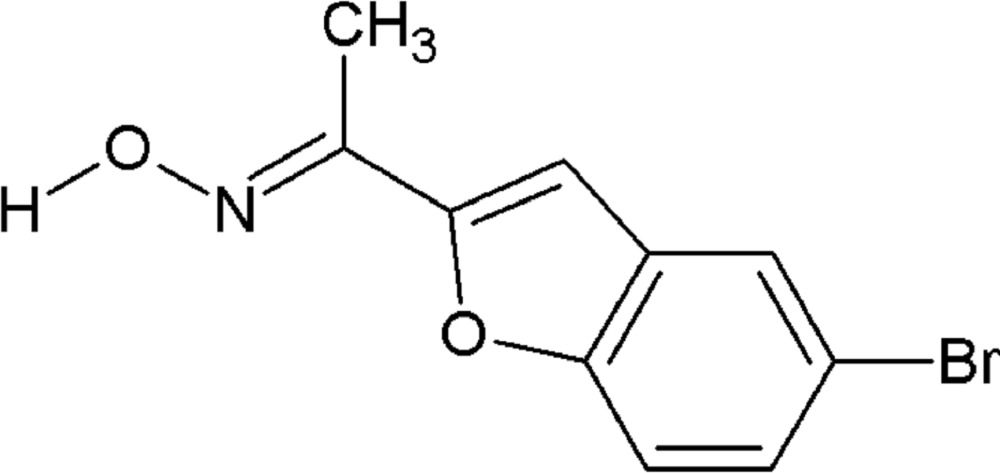


## Experimental   

### Crystal data  

C_10_H_8_BrNO_2_
*M* *_r_* = 254.08
Monoclinic, 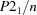

*a* = 5.9548 (6) Å
*b* = 9.4897 (10) Å
*c* = 17.2906 (19) Å
β = 96.943 (6)°
*V* = 969.91 (18) Å^3^
*Z* = 4
Mo *K*α radiation
μ = 4.21 mm^−1^
*T* = 296 K
0.32 × 0.25 × 0.21 mm

### Data collection  

Bruker APEXII diffractometer
Absorption correction: multi-scan (*SADABS*; Bruker, 2009 ▸) *T*_min_ = 0.294, *T*_max_ = 0.413
10152 measured reflections
2766 independent reflections
1937 reflections with *I* > 2σ(*I*)
*R*_int_ = 0.023

### Refinement  

*R*[*F* ^2^ > 2σ(*F* ^2^)] = 0.033
*wR*(*F* ^2^) = 0.096
*S* = 1.01
2766 reflections
129 parameters
H-atom parameters constrained
Δρ_max_ = 0.29 e Å^−3^
Δρ_min_ = −0.33 e Å^−3^

### 

Data collection: *APEX2* (Bruker, 2009 ▸); cell refinement: *SAINT-Plus* (Bruker, 2009 ▸); data reduction: *SAINT-Plus*; program(s) used to solve structure: *SHELXS97* (Sheldrick, 2008 ▸); program(s) used to refine structure: *SHELXL97* (Sheldrick, 2008 ▸); molecular graphics: *Mercury* (Macrae *et al.*, 2008 ▸); software used to prepare material for publication: *SHELXL97*.

## Supplementary Material

Crystal structure: contains datablock(s) I. DOI: 10.1107/S205698901501751X/hb7505sup1.cif

Structure factors: contains datablock(s) I. DOI: 10.1107/S205698901501751X/hb7505Isup2.hkl

Supporting information file. DOI: 10.1107/S205698901501751X/hb7505Isup3.cml

**Fig. 1.**
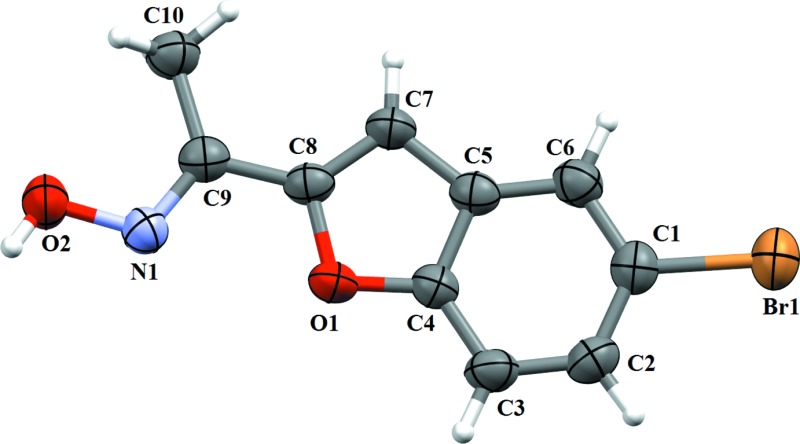
Molecular structure of the title compound, showing displacement ellipsoids drawn at the 50% probability level.

. DOI: 10.1107/S205698901501751X/hb7505fig1.tif
Mol­ecular structure of the title compound, showing displacement ellipsoids drawn at the 50% probability level.

**Fig. 2.**
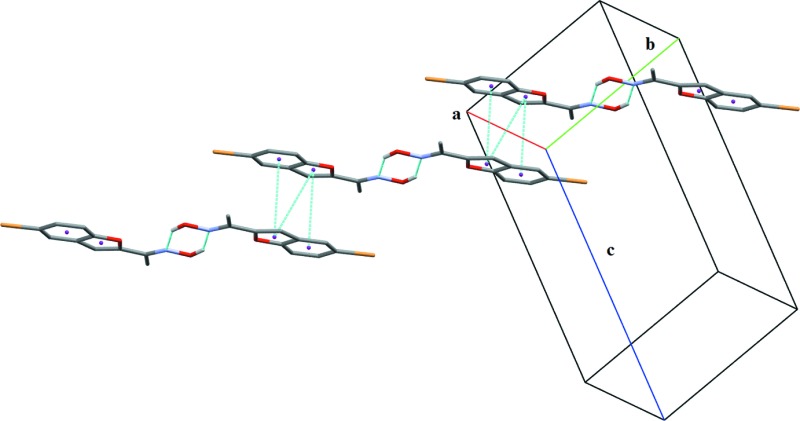
Crystal packing of the title compound displaying O—H···N and π–π interactions.

. DOI: 10.1107/S205698901501751X/hb7505fig2.tif
Crystal packing of the title compound displaying O—H⋯N and π–π inter­actions.

CCDC reference: 1425831

Additional supporting information:  crystallographic information; 3D view; checkCIF report

## Figures and Tables

**Table 1:** Hydrogen-bond geometry (, )

*D*H*A*	*D*H	H*A*	*D* *A*	*D*H*A*
O2H2*A*N1^i^	0.82	2.13	2.808(2)	140

Symmetry code: (i) 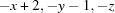.

## supplementary crystallographic information

### S1. Chemical context

The literature indicates that compounds having benzo­furan nucleus possesses versatile pharmacological activities like anifungal, anti­arrythmic, uricisuric, vasodilator and anti­migraine agent (Rida *et al.*, 2006; Manna *et al.*, 2010; Patil *et al.*, 2010; Patel *et al.*, 2006). Further, (Benzo­furan-2-yl) keoxime derivatives are known to show good anti­fungal activities (Demirayak *et al.*, 2002). In view of the above and in continuation of our efforts to study the crystal structures of benzo­furan moities (Aruna Kumar *et al.*, 2014), the title compound was synthesized and its crystal structure was determined.

### S2. Structural commentary

The title compound (I), C_10_H_8_BrNO_2_, is almost planar (r.m.s. deviation for the non-H atoms = 0.031 Å) and the conformation across the C=N bond is trans in (I) (Figure 1). In contrast to this, the conformation across the C=N bond is syn in (1Z)-1-(1-Benzo­furan-2-yl)ethanone oxime (II) (Aruna Kumar *et al.*, 2014). Further, the O atom of the benzo­furan ring is trans to the CH_3_ group in the side chain of (I), where as, in (II) (Aruna Kumar *et al.*, 2014), it is syn. The torsions in the side chain of (I) have values: O1—C8—C9—N1 = 3.3 (3)^o^, C8—C9—N1—O2 = 179.41 (17)^o^ and C7—C8—C9—C10 = 3.9 (4)^o^. The corresponding torsions in (II) have values 177.02 (16)^o^, 0.6 (3)^o^ and 178.2 (2)^o^ respectively (Aruna Kumar *et al.*, 2014).

### S3. Supra­molecular features

The crystal structure features strong O2—H2A···N1 hydrogen bonds leading into R_2_^2^(6) dimers, and these dimers are further inter­connected via two π···π inter­actions, namely cg1···cg1 and cg1···cg2 (where cg1 is the centroid of the furan ring C4/C5/C7/C8/O1 and cg2 is the centroid of the aryl ring C1—C6) (Figure 2, Table 2), the centroid-centroid separations being 3.9447 (12) Å and 3.9100 (12) Å respectively.

### S4. Synthesis and crystallization

5-bromo-2-acetyl­benzo­furan (1 g, 0.0062 mmol), hydroxyl­amine hydro­chloride (0.65 g, 0.0093 mmol) and anhydrous K_2_CO_3_ (1.29 g, 0.0093 mmol) were taken in EtOH: H_2_O (3:1, 10 mL) and refluxed for 3 h. After the completion of the reaction, the reaction mixture was poured into ice cold water. The separated white solid was filtered, washed with water and dried. It was recrystallized from EtOH.

Colourless prisms were obtained from the solvent system: ethyl acetate: methanol (4:1) by slow evapouration technique.

### S5. Refinement

Crystal data, data collection and structure refinement details are summarized in Table 1. The H atoms were positioned with idealized geometry using a riding model with C—H = 0.93–0.96 Å and O—H = 0.82 Å. The isotropic displacement parametersfor all H atoms were set to 1.2 times Ueq(Caromatic) and 1.5 times Ueq(Cmethyl, O).

### Crystal data

**Table:**

C_10_H_8_BrNO_2_	*F*(000) = 504
*M**_r_* = 254.08	Prism
Monoclinic, *P*2_1_/*n*	*D*_x_ = 1.740 Mg m^−^^3^
Hall symbol: -P 2yn	Mo *K*α radiation, λ = 0.71073 Å
*a* = 5.9548 (6) Å	Cell parameters from 125 reflections
*b* = 9.4897 (10) Å	θ = 3.5–29.9°
*c* = 17.2906 (19) Å	µ = 4.21 mm^−^^1^
β = 96.943 (6)°	*T* = 296 K
*V* = 969.91 (18) Å^3^	Prism, colourless
*Z* = 4	0.32 × 0.25 × 0.21 mm

### Data collection

**Table:**

Bruker APEXII diffractometer	1937 reflections with *I* > 2σ(*I*)
Radiation source: fine-focus sealed tube	*R*_int_ = 0.023
Graphite monochromator	θ_max_ = 29.9°, θ_min_ = 3.5°
phi and φ scans	*h* = −8→7
Absorption correction: multi-scan (*SADABS*; Bruker, 2009)	*k* = −13→10
*T*_min_ = 0.294, *T*_max_ = 0.413	*l* = −18→24
10152 measured reflections	1 standard reflections every 2 reflections
2766 independent reflections	intensity decay: 0.5%

### Refinement

**Table:**

Refinement on *F*^2^	Primary atom site location: structure-invariant direct methods
Least-squares matrix: full	Secondary atom site location: difference Fourier map
*R*[*F*^2^ > 2σ(*F*^2^)] = 0.033	Hydrogen site location: inferred from neighbouring sites
*wR*(*F*^2^) = 0.096	H-atom parameters constrained
*S* = 1.01	*w* = 1/[σ^2^(*F*_o_^2^) + (0.0563*P*)^2^ + 0.0157*P*] where *P* = (*F*_o_^2^ + 2*F*_c_^2^)/3
2766 reflections	(Δ/σ)_max_ = 0.001
129 parameters	Δρ_max_ = 0.29 e Å^−^^3^
0 restraints	Δρ_min_ = −0.33 e Å^−^^3^

### Special details

**Table:**

Geometry. All s.u.'s (except the s.u. in the dihedral angle between two l.s. planes) are estimated using the full covariance matrix. The cell s.u.'s are taken into account individually in the estimation of s.u.'s in distances, angles and torsion angles; correlations between s.u.'s in cell parameters are only used when they are defined by crystal symmetry. An approximate (isotropic) treatment of cell s.u.'s is used for estimating s.u.'s involving l.s. planes.
Refinement. Refinement of *F*^2^ against ALL reflections. The weighted *R*-factor *wR* and goodness of fit *S* are based on *F*^2^, conventional *R*-factors *R* are based on *F*, with *F* set to zero for negative *F*^2^. The threshold expression of *F*^2^ > 2σ(*F*^2^) is used only for calculating *R*-factors(gt) *etc*. and is not relevant to the choice of reflections for refinement. *R*-factors based on *F*^2^ are statistically about twice as large as those based on *F*, and *R*- factors based on ALL data will be even larger.

### Fractional atomic coordinates and isotropic or equivalent isotropic displacement parameters (Å^2^)

**Table:**

	*x*	*y*	*z*	*U*_iso_*/*U*_eq_	
C8	0.5278 (3)	−0.2686 (2)	−0.00305 (11)	0.0391 (4)	
C1	0.3056 (3)	0.0480 (2)	0.16815 (12)	0.0447 (5)	
C2	0.5215 (3)	0.0179 (2)	0.20434 (11)	0.0492 (5)	
H2	0.5745	0.0626	0.2509	0.059*	
C3	0.6569 (3)	−0.0767 (2)	0.17233 (12)	0.0492 (5)	
H3	0.8014	−0.0979	0.1961	0.059*	
C4	0.5691 (3)	−0.1394 (2)	0.10298 (11)	0.0390 (4)	
C5	0.3543 (3)	−0.1120 (2)	0.06601 (11)	0.0403 (4)	
C6	0.2169 (3)	−0.0159 (2)	0.09927 (12)	0.0470 (5)	
H6	0.0714	0.0043	0.0761	0.056*	
C7	0.3324 (3)	−0.1982 (2)	−0.00278 (12)	0.0437 (5)	
H7	0.2065	−0.2041	−0.0401	0.052*	
C9	0.6021 (3)	−0.3724 (2)	−0.05598 (11)	0.0412 (4)	
C10	0.4500 (4)	−0.4102 (3)	−0.12802 (12)	0.0513 (5)	
H10A	0.4955	−0.3594	−0.1716	0.077*	
H10B	0.2969	−0.3859	−0.1215	0.077*	
H10C	0.4598	−0.5096	−0.1373	0.077*	
N1	0.7969 (3)	−0.42793 (19)	−0.03532 (10)	0.0445 (4)	
O1	0.6789 (2)	−0.23533 (16)	0.06169 (8)	0.0440 (3)	
O2	0.8582 (2)	−0.52765 (17)	−0.08799 (9)	0.0548 (4)	
H2A	0.9720	−0.5695	−0.0688	0.082*	
Br1	0.13019 (4)	0.18428 (3)	0.214769 (13)	0.05936 (13)	

### Atomic displacement parameters (Å^2^)

**Table:**

	*U*^11^	*U*^22^	*U*^33^	*U*^12^	*U*^13^	*U*^23^
C8	0.0409 (10)	0.0392 (10)	0.0356 (10)	−0.0021 (8)	−0.0023 (7)	0.0055 (8)
C1	0.0505 (11)	0.0403 (11)	0.0447 (11)	0.0004 (8)	0.0111 (8)	0.0024 (9)
C2	0.0551 (12)	0.0525 (13)	0.0388 (11)	−0.0044 (10)	0.0009 (8)	−0.0040 (9)
C3	0.0452 (10)	0.0586 (14)	0.0412 (11)	−0.0014 (9)	−0.0058 (8)	−0.0012 (10)
C4	0.0362 (9)	0.0410 (11)	0.0394 (10)	−0.0003 (8)	0.0032 (7)	0.0031 (8)
C5	0.0401 (9)	0.0391 (11)	0.0405 (10)	−0.0005 (8)	0.0002 (7)	0.0062 (8)
C6	0.0442 (10)	0.0492 (12)	0.0464 (12)	0.0043 (9)	0.0010 (8)	0.0035 (9)
C7	0.0391 (10)	0.0498 (13)	0.0403 (11)	0.0035 (8)	−0.0037 (8)	−0.0002 (9)
C9	0.0429 (10)	0.0416 (11)	0.0384 (10)	−0.0034 (8)	0.0028 (8)	0.0051 (9)
C10	0.0520 (11)	0.0552 (14)	0.0444 (12)	0.0022 (10)	−0.0030 (9)	−0.0011 (10)
N1	0.0447 (9)	0.0462 (10)	0.0420 (9)	0.0057 (7)	0.0028 (7)	−0.0024 (7)
O1	0.0379 (7)	0.0496 (8)	0.0420 (7)	0.0041 (6)	−0.0052 (5)	−0.0014 (6)
O2	0.0562 (9)	0.0591 (10)	0.0493 (8)	0.0142 (7)	0.0066 (6)	−0.0069 (7)
Br1	0.0661 (2)	0.05433 (19)	0.06028 (19)	0.00532 (9)	0.01851 (12)	−0.00638 (10)

### Geometric parameters (Å, º)

**Table:**

C8—C7	1.343 (3)	C5—C6	1.394 (3)
C8—O1	1.385 (2)	C5—C7	1.437 (3)
C8—C9	1.449 (3)	C6—H6	0.9300
C1—C6	1.383 (3)	C7—H7	0.9300
C1—C2	1.390 (3)	C9—N1	1.285 (3)
C1—Br1	1.901 (2)	C9—C10	1.493 (3)
C2—C3	1.367 (3)	C10—H10A	0.9600
C2—H2	0.9300	C10—H10B	0.9600
C3—C4	1.383 (3)	C10—H10C	0.9600
C3—H3	0.9300	N1—O2	1.392 (2)
C4—O1	1.371 (2)	O2—H2A	0.8200
C4—C5	1.384 (2)		
			
C7—C8—O1	111.24 (18)	C1—C6—C5	117.40 (17)
C7—C8—C9	132.16 (16)	C1—C6—H6	121.3
O1—C8—C9	116.56 (17)	C5—C6—H6	121.3
C6—C1—C2	122.23 (19)	C8—C7—C5	107.11 (16)
C6—C1—Br1	119.44 (15)	C8—C7—H7	126.4
C2—C1—Br1	118.33 (15)	C5—C7—H7	126.4
C3—C2—C1	120.75 (18)	N1—C9—C8	115.94 (16)
C3—C2—H2	119.6	N1—C9—C10	124.7 (2)
C1—C2—H2	119.6	C8—C9—C10	119.31 (17)
C2—C3—C4	117.00 (18)	C9—C10—H10A	109.5
C2—C3—H3	121.5	C9—C10—H10B	109.5
C4—C3—H3	121.5	H10A—C10—H10B	109.5
O1—C4—C3	125.70 (16)	C9—C10—H10C	109.5
O1—C4—C5	110.89 (16)	H10A—C10—H10C	109.5
C3—C4—C5	123.4 (2)	H10B—C10—H10C	109.5
C4—C5—C6	119.22 (18)	C9—N1—O2	113.35 (16)
C4—C5—C7	105.18 (17)	C4—O1—C8	105.58 (15)
C6—C5—C7	135.61 (16)	N1—O2—H2A	109.5
			
C6—C1—C2—C3	0.5 (3)	C9—C8—C7—C5	178.2 (2)
Br1—C1—C2—C3	−178.43 (16)	C4—C5—C7—C8	−0.3 (2)
C1—C2—C3—C4	0.2 (3)	C6—C5—C7—C8	179.5 (2)
C2—C3—C4—O1	179.3 (2)	C7—C8—C9—N1	−174.5 (2)
C2—C3—C4—C5	−0.6 (3)	O1—C8—C9—N1	3.3 (3)
O1—C4—C5—C6	−179.65 (18)	C7—C8—C9—C10	3.9 (4)
C3—C4—C5—C6	0.3 (3)	O1—C8—C9—C10	−178.38 (19)
O1—C4—C5—C7	0.2 (2)	C8—C9—N1—O2	179.41 (17)
C3—C4—C5—C7	−179.9 (2)	C10—C9—N1—O2	1.1 (3)
C2—C1—C6—C5	−0.9 (3)	C3—C4—O1—C8	−179.9 (2)
Br1—C1—C6—C5	178.09 (15)	C5—C4—O1—C8	0.0 (2)
C4—C5—C6—C1	0.4 (3)	C7—C8—O1—C4	−0.3 (2)
C7—C5—C6—C1	−179.3 (2)	C9—C8—O1—C4	−178.48 (16)
O1—C8—C7—C5	0.4 (2)		

### Hydrogen-bond geometry (Å, º)

**Table:**

*D*—H···*A*	*D*—H	H···*A*	*D*···*A*	*D*—H···*A*
O2—H2*A*···N1^i^	0.82	2.13	2.808 (2)	140

Symmetry code: (i) −*x*+2, −*y*−1, −*z*.
