# Supplementary figures and images for: Crystal structure of 1-(5-bromo-1-benzo­furan-2-yl)ethanone oxime
Source: Acta Crystallogr E Crystallogr Commun. 2015 Sep 26;71(Pt 10):o773–4. doi: 10.1107/S205698901501751X (PMC4647387; doi:10.1107/S205698901501751X)

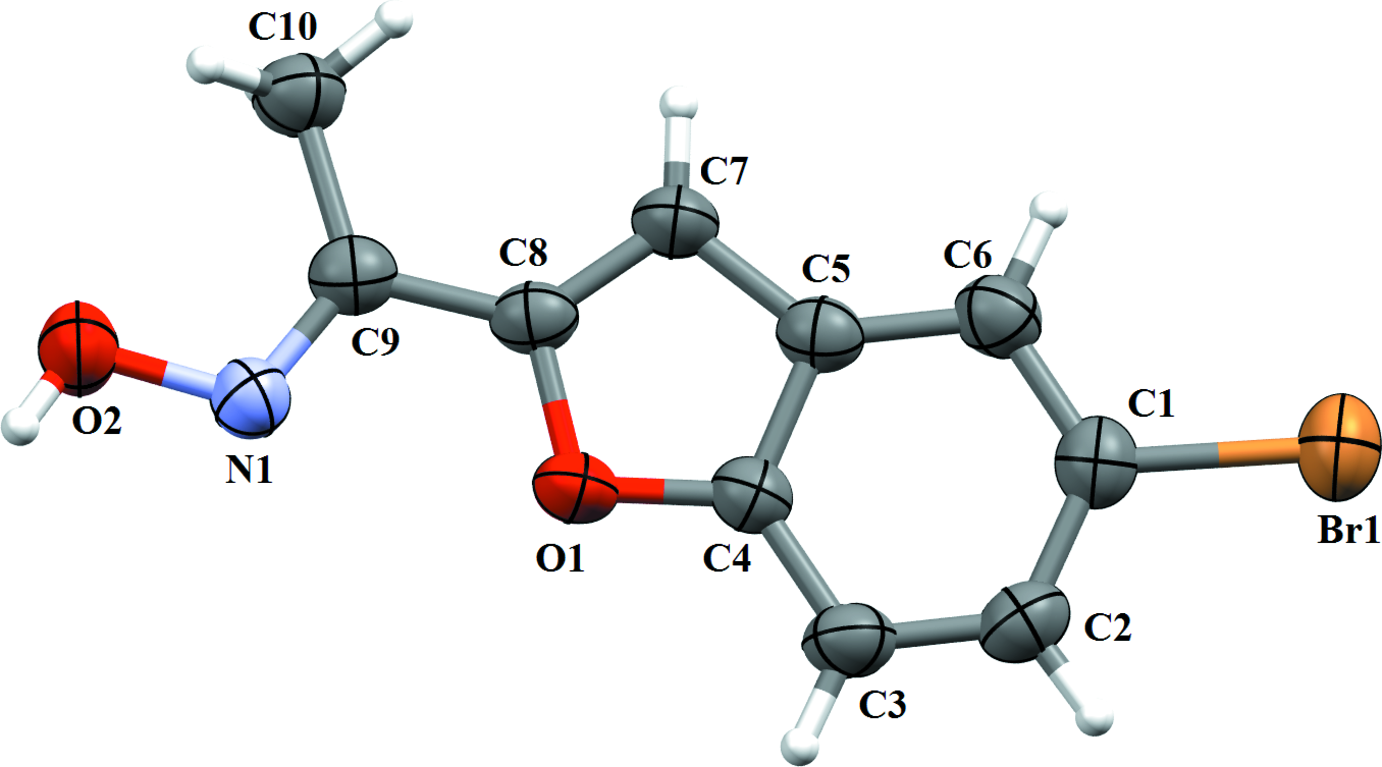

Supplement: Supplementary file 4 [file e-71-0o773-fig1.tif]

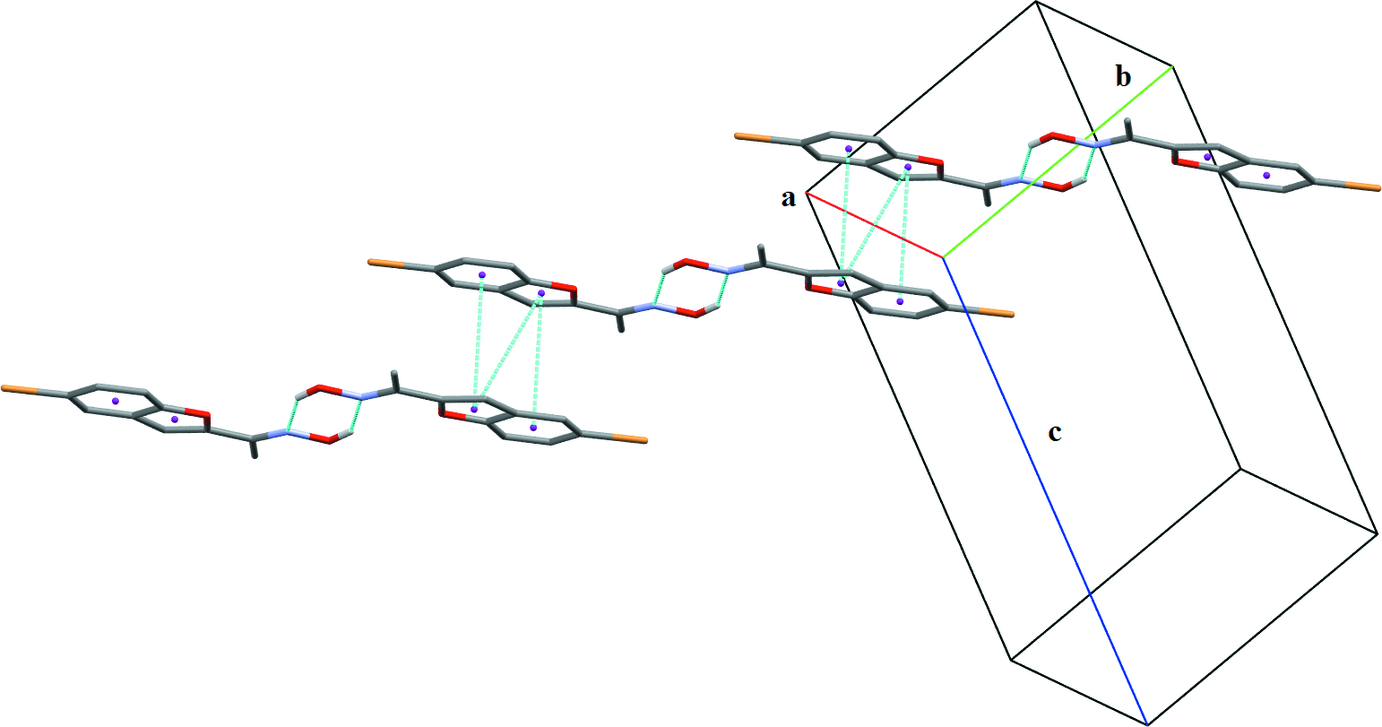

Supplement: Supplementary file 5 [file e-71-0o773-fig2.tif]
